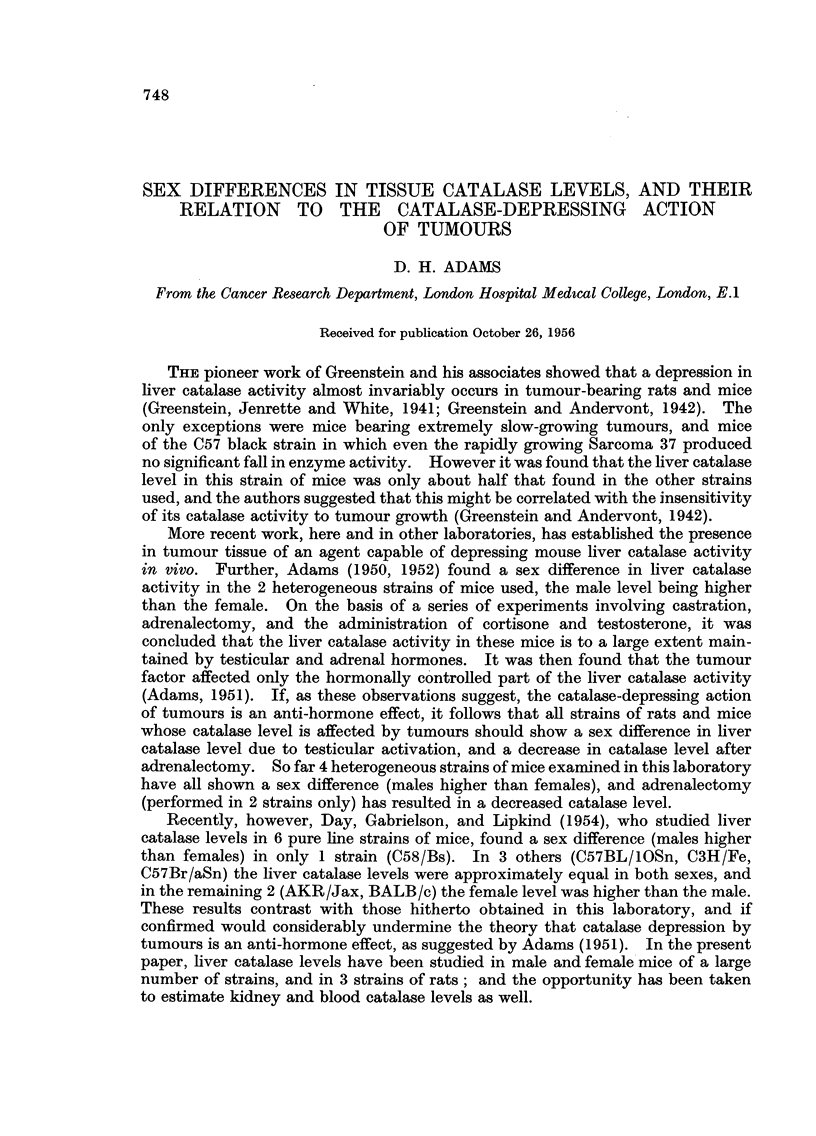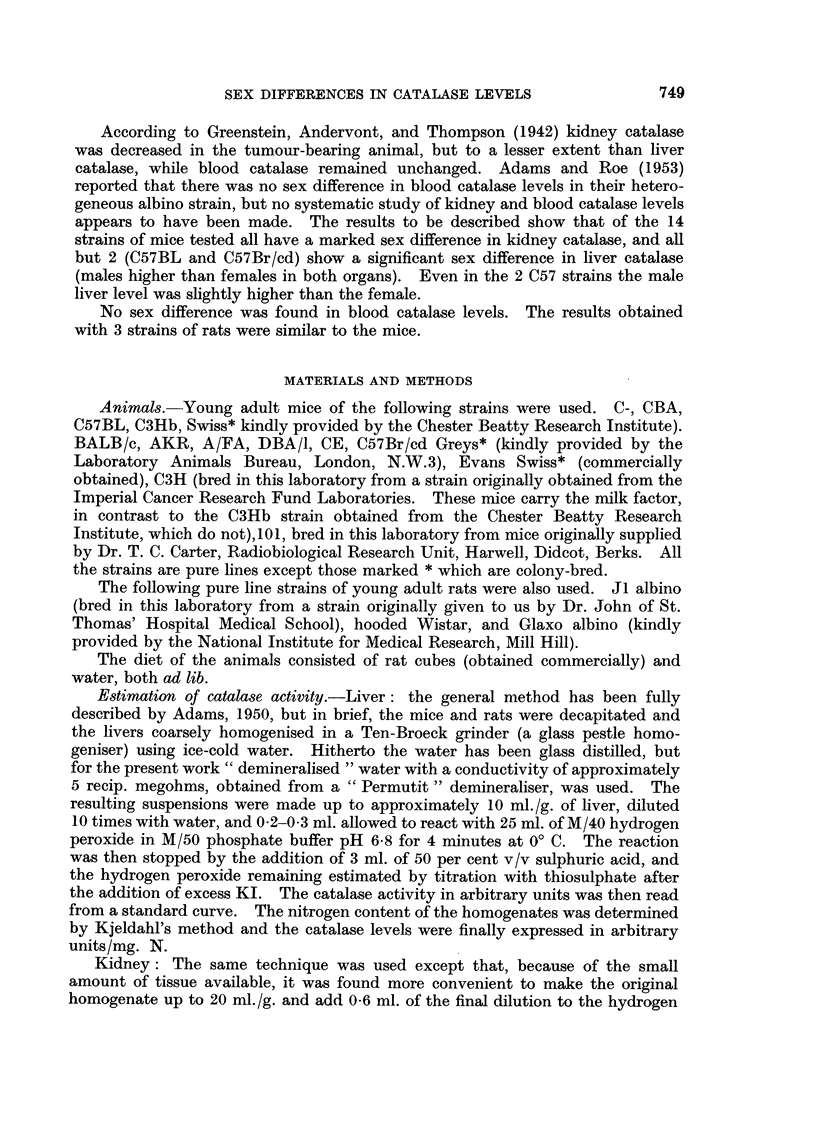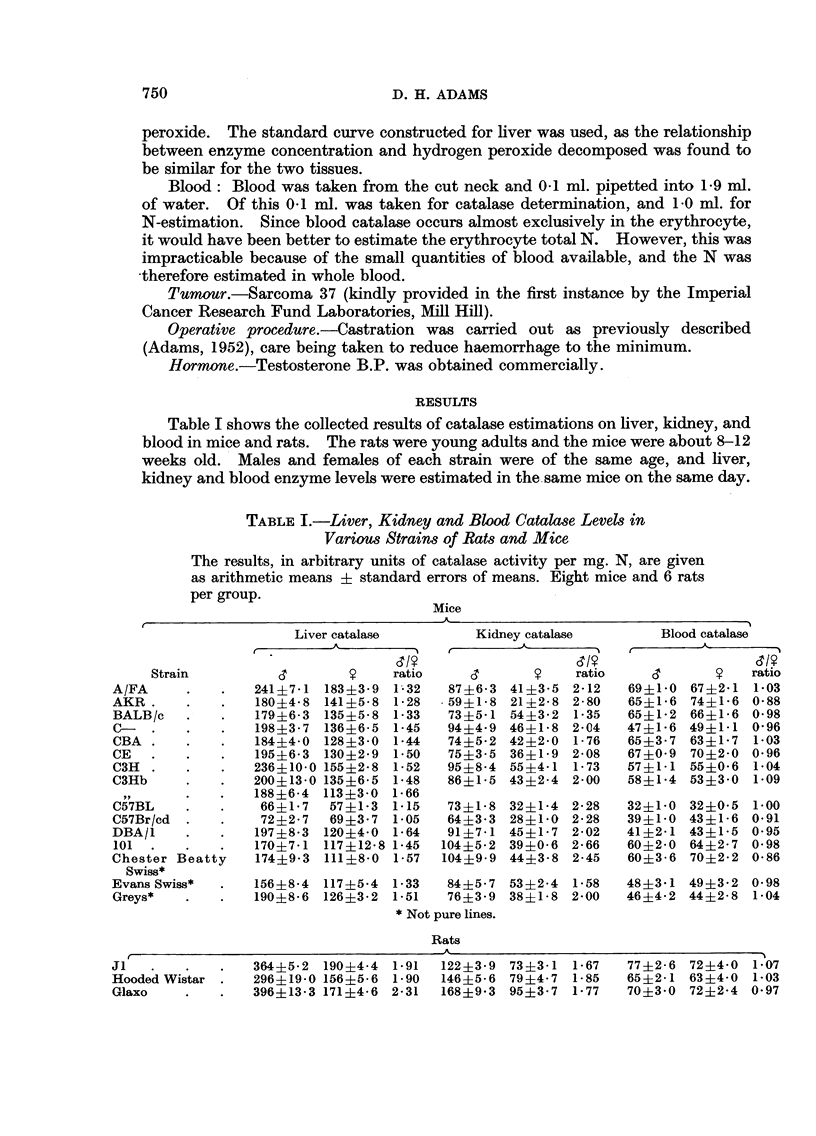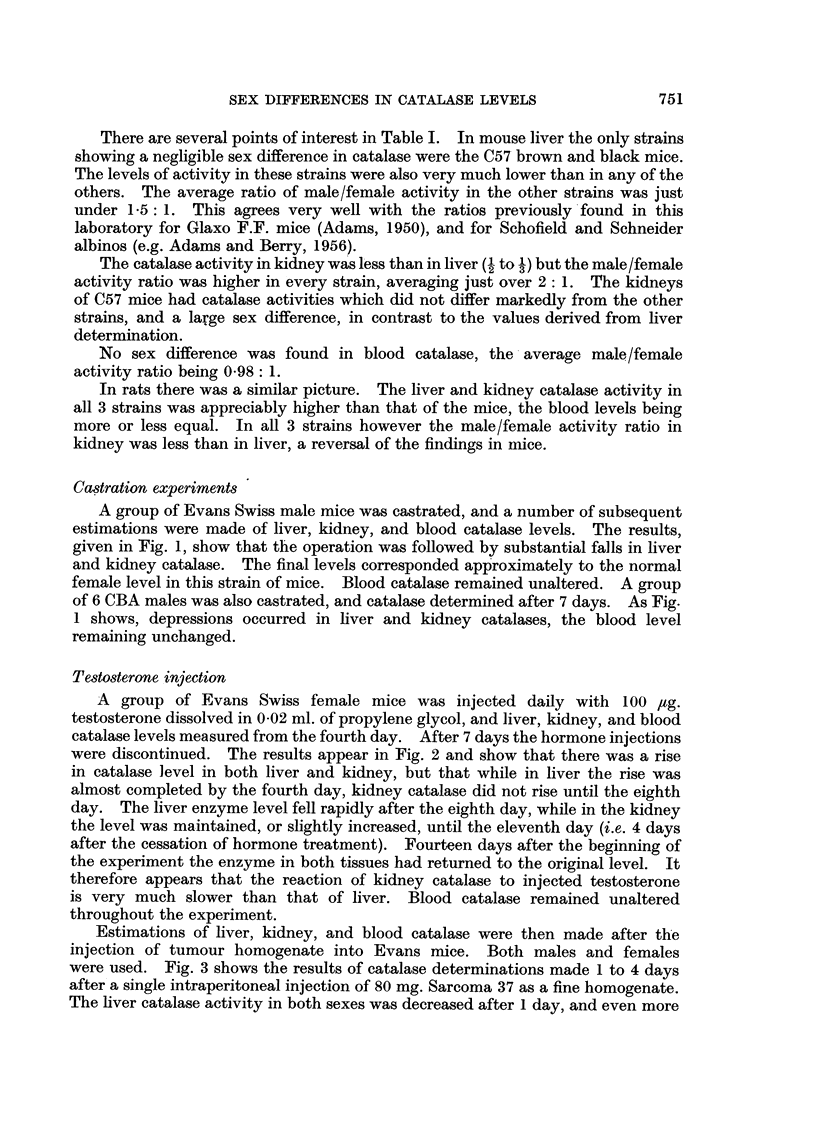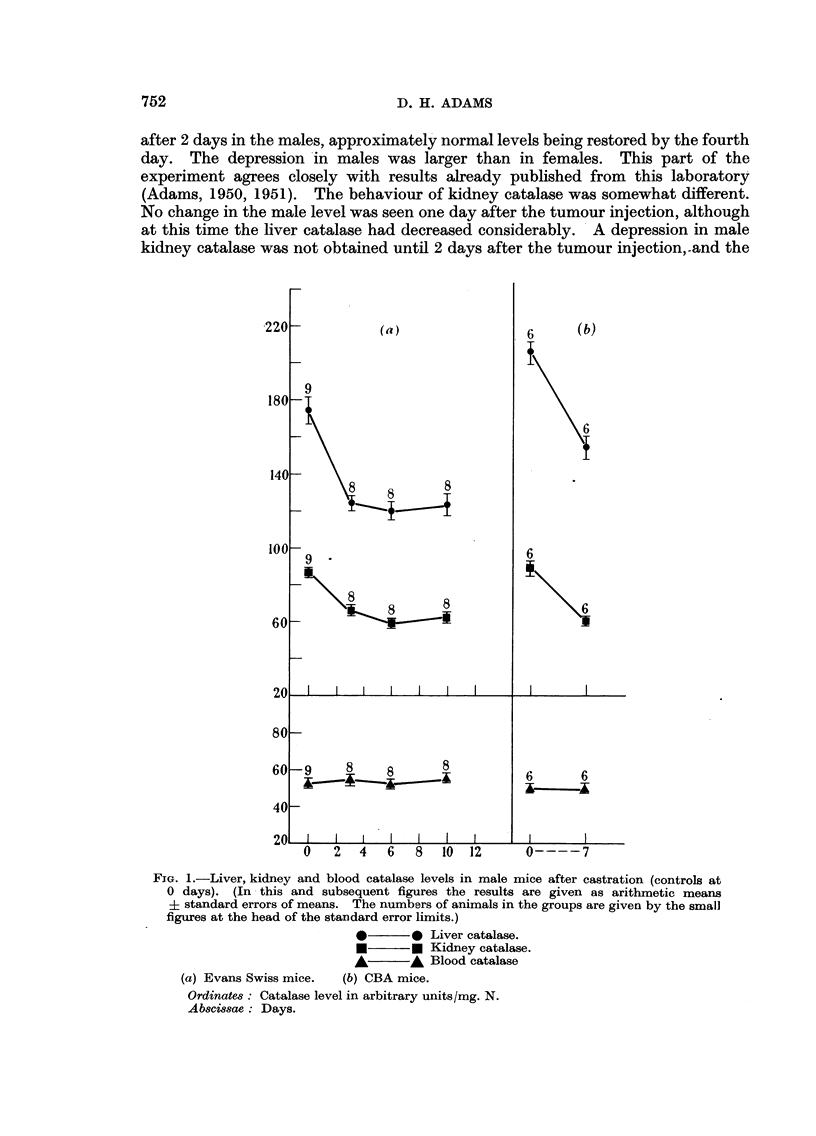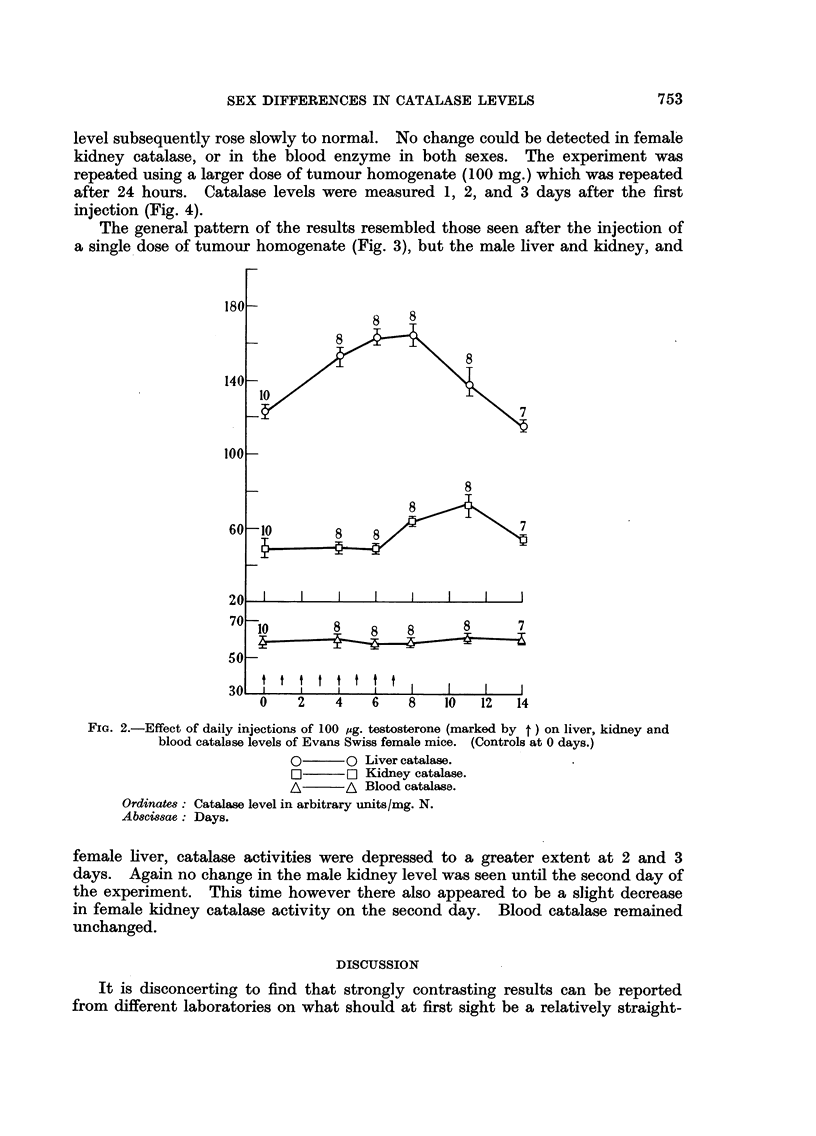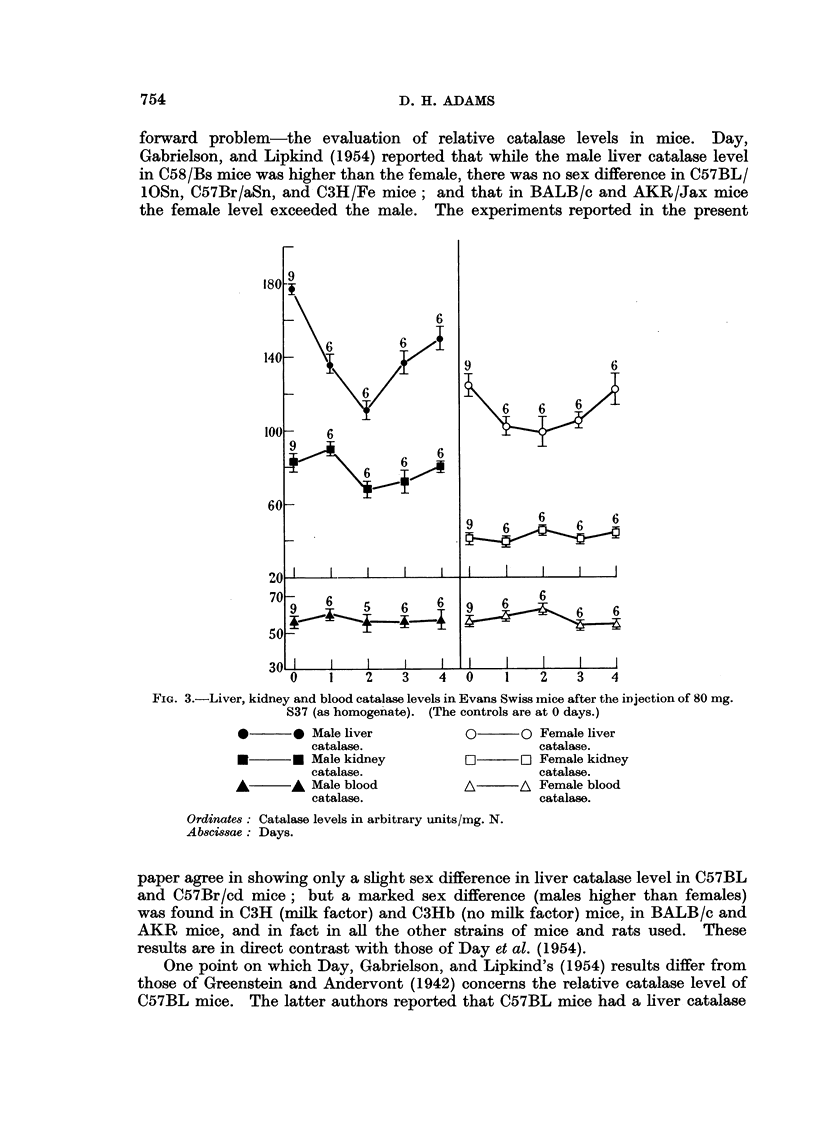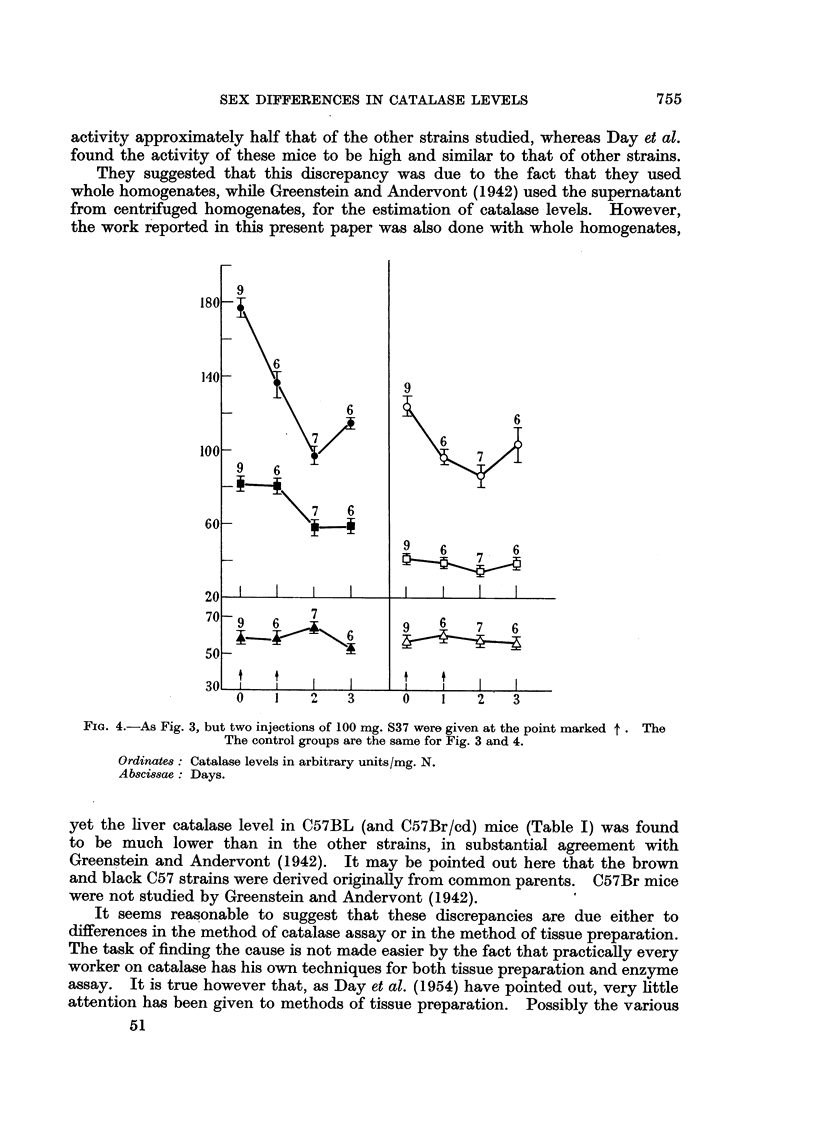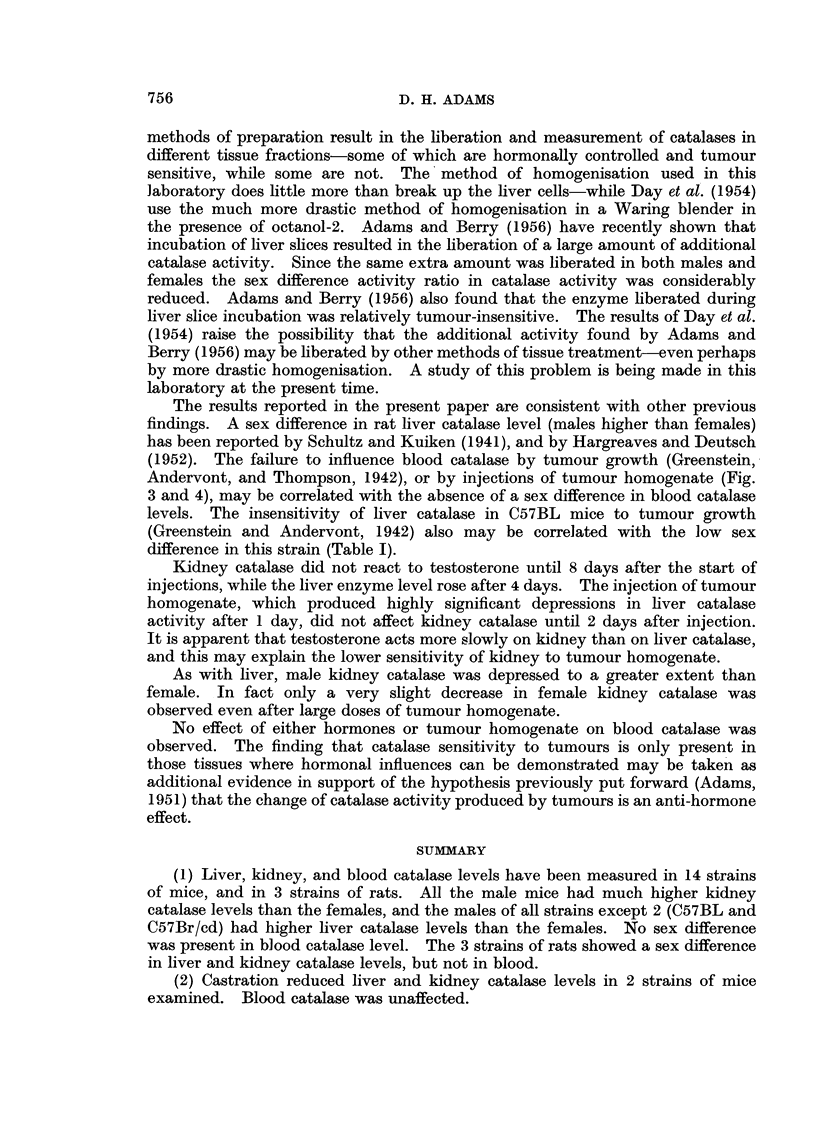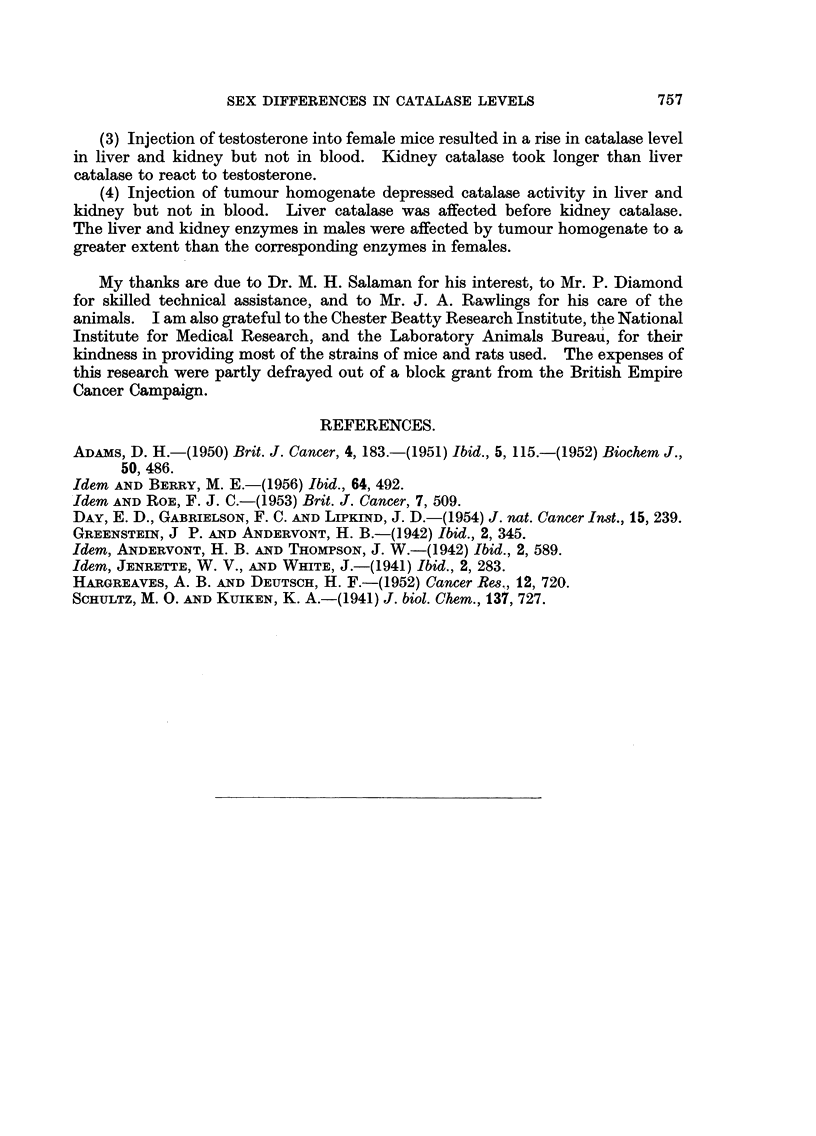# Sex Differences in Tissue Catalase Levels, and Their Relation to the Catalase-Depressing action of Tumours

**DOI:** 10.1038/bjc.1956.91

**Published:** 1956-12

**Authors:** D. H. Adams


### SEX DIFFERENCES IN TISSUE CATALASE LEVELS, AND THEIR RELATION TO THE CATALASE-DEPRESSING ACTION OF TUMOURS

D. H. ADAMS

*From the Cancer Research Department, London Hospital Medical College, London, E.*1

Received for publication October 26, 1956

THE pioneer work of Greenstein and his associates showed that a depression in liver catalase activity almost invariably occurs in tumour-bearing rats and mice (Greenstein, Jenrette and White, 1941; Greenstein and Andervont, 1942). The only exceptions were mice bearing extremely slow-growing tumours, and mice of the C57 black strain in which even the rapidly growing Sarcoma 37 produced no significant fall in enzyme activity. However it was found that the liver catalase level in this strain of mice was only about half that found in the other strains used, and the authors suggested that this might be correlated with the insensitivity of its catalase activity to tumour growth (Greenstein and Andervont, 1942).

More recent work, here and in other laboratories, has established the presence in tumour tissue of an agent capable of depressing mouse liver catalase activity *in vivo*. Further, Adams (1950, 1952) found a sex difference in liver catalase activity in the 2 heterogeneous strains of mice used, the male level being higher than the female. On the basis of a series of experiments involving castration, adrenalectomy, and the administration of cortisone and testosterone, it was concluded that the liver catalase activity in these mice is to a large extent maintained by testicular and adrenal hormones. It was then found that the tumour factor affected only the hormonally controlled part of the liver catalase activity (Adams, 1951). If, as these observations suggest, the catalase-depressing action of tumours is an anti-hormone effect, it follows that all strains of rats and mice whose catalase level is affected by tumours should show a sex difference in liver catalase level due to testicular activation, and a decrease in catalase level after adrenalectomy. So far 4 heterogeneous strains of mice examined in this laboratory have all shown a sex difference (males higher than females), and adrenalectomy (performed in 2 strains only) has resulted in a decreased catalase level.

Recently, however, Day, Gabrielson, and Lipkind (1954), who studied liver catalase levels in 6 pure line strains of mice, found a sex difference (males higher than females) in only 1 strain (C58/Bs). In 3 others (C57BL/1OSn, C3H/Fe, C57Br/aSn) the liver catalase levels were approximately equal in both sexes, and in the remaining 2 (AKR/Jax, BALB/c) the female level was higher than the male. These results contrast with those hitherto obtained in this laboratory, and if confirmed would considerably undermine the theory that catalase depression by tumours is an anti-hormone effect, as suggested by Adams (1951). In the present paper, liver catalase levels have been studied in male and female mice of a large number of strains, and in 3 strains of rats; and the opportunity has been taken to estimate kidney and blood catalase levels as well.

According to Greenstein, Andervont, and Thompson (1942) kidney catalase was decreased in the tumour-bearing animal, but to a lesser extent than liver catalase, while blood catalase remained unchanged. Adams and Roe (1953) reported that there was no sex difference in blood catalase levels in their heterogeneous albino strain, but no systematic study of kidney and blood catalase levels appears to have been made. The results to be described show that of the 14 strains of mice tested all have a marked sex difference in kidney catalase, and all but 2 (C57BL and C57Br/cd) show a significant sex difference in liver catalase (males higher than females in both organs). Even in the 2 C57 strains the male liver level was slightly higher than the female.

No sex difference was found in blood catalase levels. The results obtained with 3 strains of rats were similar to the mice.

### MATERIALS AND METHODS

*Animals.*—Young adult mice of the following strains were used. C-, CBA, C57BL, C3Hb, Swiss* kindly provided by the Chester Beatty Research Institute). BALB/c, AKR, A/FA, DBA/1, CE, C57Br/cd Greys* (kindly provided by the Laboratory Animals Bureau, London, N.W.3), Evans Swiss* (commercially obtained), C3H (bred in this laboratory from a strain originally obtained from the Imperial Cancer Research Fund Laboratories. These mice carry the milk factor, in contrast to the C3Hb strain obtained from the Chester Beatty Research Institute, which do not),101, bred in this laboratory from mice originally supplied by Dr. T. C. Carter, Radiobiological Research Unit, Harwell, Didcot, Berks. All the strains are pure lines except those marked * which are colony-bred.

The following pure line strains of young adult rats were also used. J1 albino (bred in this laboratory from a strain originally given to us by Dr. John of St. Thomas' Hospital Medical School), hooded Wistar, and Glaxo albino (kindly provided by the National Institute for Medical Research, Mill Hill).

The diet of the animals consisted of rat cubes (obtained commercially) and water, both *ad lib.*

*Estimation of catalase activity.*—Liver : the general method has been fully described by Adams, 1950, but in brief, the mice and rats were decapitated and the livers coarsely homogenised in a Ten-Broeck grinder (a glass pestle homogeniser) using ice-cold water. Hitherto the water has been glass distilled, but for the present work " demineralised " water with a conductivity of approximately 5 recip. megohms, obtained from a " Permutit " demineraliser, was used. The resulting suspensions were made up to approximately 10 ml./g. of liver, diluted 10 times with water, and 0·2–0·3 ml. allowed to react with 25 ml. of M/40 hydrogen peroxide in M/50 phosphate buffer pH 6·8 for 4 minutes at 0° C. The reaction was then stopped by the addition of 3 ml. of 50 per cent v/v sulphuric acid, and the hydrogen peroxide remaining estimated by titration with thiosulphate after the addition of excess KI. The catalase activity in arbitrary units was then read from a standard curve. The nitrogen content of the homogenates was determined by Kjeldahl's method and the catalase levels were finally expressed in arbitrary units/mg. N.

Kidney : The same technique was used except that, because of the small amount of tissue available, it was found more convenient to make the original homogenate up to 20 ml./g. and add 0·6 ml. of the final dilution to the hydrogen peroxide. The standard curve constructed for liver was used, as the relationship between enzyme concentration and hydrogen peroxide decomposed was found to be similar for the two tissues.

Blood : Blood was taken from the cut neck and 0·1 ml. pipetted into 1·9 ml. of water. Of this 0·1 ml. was taken for catalase determination, and 1·0 ml. for N-estimation. Since blood catalase occurs almost exclusively in the erythrocyte, it would have been better to estimate the erythrocyte total N. However, this was impracticable because of the small quantities of blood available, and the N was therefore estimated in whole blood.

*Tumour.*—Sarcoma 37 (kindly provided in the first instance by the Imperial Cancer Research Fund Laboratories, Mill Hill).

*Operative procedure.*—Castration was carried out as previously described (Adams, 1952), care being taken to reduce haemorrhage to the minimum.

*Hormone.*—Testosterone B.P. was obtained commercially.

### RESULTS

Table I shows the collected results of catalase estimations on liver, kidney, and blood in mice and rats. The rats were young adults and the mice were about 8–12 weeks old. Males and females of each strain were of the same age, and liver, kidney and blood enzyme levels were estimated in the same mice on the same day.

TABLE I.—*Liver, Kidney and Blood Catalase Levels in Various Strains of Rats and Mice*

The results, in arbitrary units of catalase activity per mg. N, are given as arithmetic means ± standard errors of means. Eight mice and 6 rats per group.

| | Liver catalase | | | Kidney catalase | | | Blood catalase | | |
|---|---|---|---|---|---|---|---|---|---|
| Strain | ♂ | ♀ | ♂/♀ ratio | ♂ | ♀ | ♂/♀ ratio | ♂ | ♀ | ♂/♀ ratio |
| **Mice** | | | | | | | | | |
| A/FA | 241±7·1 | 183±3·9 | 1·32 | 87±6·3 | 41±3·5 | 2·12 | 69±1·0 | 67±2·1 | 1·03 |
| AKR | 180±4·8 | 141±5·8 | 1·28 | 59±1·8 | 21±2·8 | 2·80 | 65±1·6 | 74±1·6 | 0·88 |
| BALB/c | 179±6·3 | 135±5·8 | 1·33 | 73±5·1 | 54±3·2 | 1·35 | 65±1·2 | 66±1·6 | 0·98 |
| C— | 198±3·7 | 136±6·5 | 1·45 | 94±4·9 | 46±1·8 | 2·04 | 47±1·6 | 49±1·1 | 0·96 |
| CBA | 184±4·0 | 128±3·0 | 1·44 | 74±5·2 | 42±2·0 | 1·76 | 65±3·7 | 63±1·7 | 1·03 |
| CE | 195±6·3 | 130±2·9 | 1·50 | 75±3·5 | 36±1·9 | 2·08 | 67±0·9 | 70±2·0 | 0·96 |
| C3H | 236±10·0 | 155±2·8 | 1·52 | 95±8·4 | 55±4·1 | 1·73 | 57±1·1 | 55±0·6 | 1·04 |
| C3Hb | 200±13·0 | 135±6·5 | 1·48 | 86±1·5 | 43±2·4 | 2·00 | 58±1·4 | 53±3·0 | 1·09 |
| „ | 188±6·4 | 113±3·0 | 1·66 | | | | | | |
| C57BL | 66±1·7 | 57±1·3 | 1·15 | 73±1·8 | 32±1·4 | 2·28 | 32±1·0 | 32±0·5 | 1·00 |
| C57Br/cd | 72±2·7 | 69±3·7 | 1·05 | 64±3·3 | 28±1·0 | 2·28 | 39±1·0 | 43±1·6 | 0·91 |
| DBA/1 | 197±8·3 | 120±4·0 | 1·64 | 91±7·1 | 45±1·7 | 2·02 | 41±2·1 | 43±1·5 | 0·95 |
| 101 | 170±7·1 | 117±12·8 | 1·45 | 104±5·2 | 39±0·6 | 2·66 | 60±2·0 | 64±2·7 | 0·98 |
| Chester Beatty Swiss* | 174±9·3 | 111±8·0 | 1·57 | 104±9·9 | 44±3·8 | 2·45 | 60±3·6 | 70±2·2 | 0·86 |
| Evans Swiss* | 156±8·4 | 117±5·4 | 1·33 | 84±5·7 | 53±2·4 | 1·58 | 48±3·1 | 49±3·2 | 0·98 |
| Greys* | 190±8·6 | 126±3·2 | 1·51 | 76±3·9 | 38±1·8 | 2·00 | 46±4·2 | 44±2·8 | 1·04 |
| **Rats** | | | | | | | | | |
| J1 | 364±5·2 | 190±4·4 | 1·91 | 122±3·9 | 73±3·1 | 1·67 | 77±2·6 | 72±4·0 | 1·07 |
| Hooded Wistar | 296±19·0 | 156±5·6 | 1·90 | 146±5·6 | 79±4·7 | 1·85 | 65±2·1 | 63±4·0 | 1·03 |
| Glaxo | 396±13·3 | 171±4·6 | 2·31 | 168±9·3 | 95±3·7 | 1·77 | 70±3·0 | 72±2·4 | 0·97 |

* Not pure lines.

There are several points of interest in Table I. In mouse liver the only strains showing a negligible sex difference in catalase were the C57 brown and black mice. The levels of activity in these strains were also very much lower than in any of the others. The average ratio of male/female activity in the other strains was just under 1·5 : 1. This agrees very well with the ratios previously found in this laboratory for Glaxo F.F. mice (Adams, 1950), and for Schofield and Schneider albinos (e.g. Adams and Berry, 1956).

The catalase activity in kidney was less than in liver ($\frac{1}{2}$ to $\frac{1}{3}$) but the male/female activity ratio was higher in every strain, averaging just over 2 : 1. The kidneys of C57 mice had catalase activities which did not differ markedly from the other strains, and a large sex difference, in contrast to the values derived from liver determination.

No sex difference was found in blood catalase, the average male/female activity ratio being 0·98 : 1.

In rats there was a similar picture. The liver and kidney catalase activity in all 3 strains was appreciably higher than that of the mice, the blood levels being more or less equal. In all 3 strains however the male/female activity ratio in kidney was less than in liver, a reversal of the findings in mice.

*Castration experiments*

A group of Evans Swiss male mice was castrated, and a number of subsequent estimations were made of liver, kidney, and blood catalase levels. The results, given in Fig. 1, show that the operation was followed by substantial falls in liver and kidney catalase. The final levels corresponded approximately to the normal female level in this strain of mice. Blood catalase remained unaltered. A group of 6 CBA males was also castrated, and catalase determined after 7 days. As Fig. 1 shows, depressions occurred in liver and kidney catalases, the blood level remaining unchanged.

*Testosterone injection*

A group of Evans Swiss female mice was injected daily with 100 $\mu$g. testosterone dissolved in 0·02 ml. of propylene glycol, and liver, kidney, and blood catalase levels measured from the fourth day. After 7 days the hormone injections were discontinued. The results appear in Fig. 2 and show that there was a rise in catalase level in both liver and kidney, but that while in liver the rise was almost completed by the fourth day, kidney catalase did not rise until the eighth day. The liver enzyme level fell rapidly after the eighth day, while in the kidney the level was maintained, or slightly increased, until the eleventh day (*i.e.* 4 days after the cessation of hormone treatment). Fourteen days after the beginning of the experiment the enzyme in both tissues had returned to the original level. It therefore appears that the reaction of kidney catalase to injected testosterone is very much slower than that of liver. Blood catalase remained unaltered throughout the experiment.

Estimations of liver, kidney, and blood catalase were then made after the injection of tumour homogenate into Evans mice. Both males and females were used. Fig. 3 shows the results of catalase determinations made 1 to 4 days after a single intraperitoneal injection of 80 mg. Sarcoma 37 as a fine homogenate. The liver catalase activity in both sexes was decreased after 1 day, and even more after 2 days in the males, approximately normal levels being restored by the fourth day. The depression in males was larger than in females. This part of the experiment agrees closely with results already published from this laboratory (Adams, 1950, 1951). The behaviour of kidney catalase was somewhat different. No change in the male level was seen one day after the tumour injection, although at this time the liver catalase had decreased considerably. A depression in male kidney catalase was not obtained until 2 days after the tumour injection, and the

Fig. 1.—Liver, kidney and blood catalase levels in male mice after castration (controls at 0 days). (In this and subsequent figures the results are given as arithmetic means ± standard errors of means. The numbers of animals in the groups are given by the small figures at the head of the standard error limits.)

●——● Liver catalase.
■——■ Kidney catalase.
▲——▲ Blood catalase

(*a*) Evans Swiss mice. (*b*) CBA mice.

*Ordinates :* Catalase level in arbitrary units/mg. N.
*Abscissae :* Days.

level subsequently rose slowly to normal. No change could be detected in female kidney catalase, or in the blood enzyme in both sexes. The experiment was repeated using a larger dose of tumour homogenate (100 mg.) which was repeated after 24 hours. Catalase levels were measured 1, 2, and 3 days after the first injection (Fig. 4).

The general pattern of the results resembled those seen after the injection of a single dose of tumour homogenate (Fig. 3), but the male liver and kidney, and

FIG. 2.—Effect of daily injections of 100 $\mu$g. testosterone (marked by ↑) on liver, kidney and blood catalase levels of Evans Swiss female mice. (Controls at 0 days.)

○———○ Liver catalase.
□———□ Kidney catalase.
△———△ Blood catalase.

*Ordinates :* Catalase level in arbitrary units/mg. N.
*Abscissae :* Days.

female liver, catalase activities were depressed to a greater extent at 2 and 3 days. Again no change in the male kidney level was seen until the second day of the experiment. This time however there also appeared to be a slight decrease in female kidney catalase activity on the second day. Blood catalase remained unchanged.

### DISCUSSION

It is disconcerting to find that strongly contrasting results can be reported from different laboratories on what should at first sight be a relatively straightforward problem—the evaluation of relative catalase levels in mice. Day, Gabrielson, and Lipkind (1954) reported that while the male liver catalase level in C58/Bs mice was higher than the female, there was no sex difference in C57BL/10Sn, C57Br/aSn, and C3H/Fe mice ; and that in BALB/c and AKR/Jax mice the female level exceeded the male. The experiments reported in the present

FIG. 3.—Liver, kidney and blood catalase levels in Evans Swiss mice after the injection of 80 mg. S37 (as homogenate). (The controls are at 0 days.)

●——● Male liver catalase. ○——○ Female liver catalase.

■——■ Male kidney catalase. □——□ Female kidney catalase.

▲——▲ Male blood catalase. △——△ Female blood catalase.

*Ordinates :* Catalase levels in arbitrary units/mg. N.
*Abscissae :* Days.

paper agree in showing only a slight sex difference in liver catalase level in C57BL and C57Br/cd mice ; but a marked sex difference (males higher than females) was found in C3H (milk factor) and C3Hb (no milk factor) mice, in BALB/c and AKR mice, and in fact in all the other strains of mice and rats used. These results are in direct contrast with those of Day *et al.* (1954).

One point on which Day, Gabrielson, and Lipkind's (1954) results differ from those of Greenstein and Andervont (1942) concerns the relative catalase level of C57BL mice. The latter authors reported that C57BL mice had a liver catalase activity approximately half that of the other strains studied, whereas Day *et al.* found the activity of these mice to be high and similar to that of other strains.

They suggested that this discrepancy was due to the fact that they used whole homogenates, while Greenstein and Andervont (1942) used the supernatant from centrifuged homogenates, for the estimation of catalase levels. However, the work reported in this present paper was also done with whole homogenates,

FIG. 4.—As Fig. 3, but two injections of 100 mg. S37 were given at the point marked ↑. The The control groups are the same for Fig. 3 and 4.

*Ordinates :* Catalase levels in arbitrary units/mg. N.
*Abscissae :* Days.

yet the liver catalase level in C57BL (and C57Br/cd) mice (Table I) was found to be much lower than in the other strains, in substantial agreement with Greenstein and Andervont (1942). It may be pointed out here that the brown and black C57 strains were derived originally from common parents. C57Br mice were not studied by Greenstein and Andervont (1942).

It seems reasonable to suggest that these discrepancies are due either to differences in the method of catalase assay or in the method of tissue preparation. The task of finding the cause is not made easier by the fact that practically every worker on catalase has his own techniques for both tissue preparation and enzyme assay. It is true however that, as Day *et al.* (1954) have pointed out, very little attention has been given to methods of tissue preparation. Possibly the various methods of preparation result in the liberation and measurement of catalases in different tissue fractions—some of which are hormonally controlled and tumour sensitive, while some are not. The method of homogenisation used in this laboratory does little more than break up the liver cells—while Day *et al.* (1954) use the much more drastic method of homogenisation in a Waring blender in the presence of octanol-2. Adams and Berry (1956) have recently shown that incubation of liver slices resulted in the liberation of a large amount of additional catalase activity. Since the same extra amount was liberated in both males and females the sex difference activity ratio in catalase activity was considerably reduced. Adams and Berry (1956) also found that the enzyme liberated during liver slice incubation was relatively tumour-insensitive. The results of Day *et al.* (1954) raise the possibility that the additional activity found by Adams and Berry (1956) may be liberated by other methods of tissue treatment—even perhaps by more drastic homogenisation. A study of this problem is being made in this laboratory at the present time.

The results reported in the present paper are consistent with other previous findings. A sex difference in rat liver catalase level (males higher than females) has been reported by Schultz and Kuiken (1941), and by Hargreaves and Deutsch (1952). The failure to influence blood catalase by tumour growth (Greenstein, Andervont, and Thompson, 1942), or by injections of tumour homogenate (Fig. 3 and 4), may be correlated with the absence of a sex difference in blood catalase levels. The insensitivity of liver catalase in C57BL mice to tumour growth (Greenstein and Andervont, 1942) also may be correlated with the low sex difference in this strain (Table I).

Kidney catalase did not react to testosterone until 8 days after the start of injections, while the liver enzyme level rose after 4 days. The injection of tumour homogenate, which produced highly significant depressions in liver catalase activity after 1 day, did not affect kidney catalase until 2 days after injection. It is apparent that testosterone acts more slowly on kidney than on liver catalase, and this may explain the lower sensitivity of kidney to tumour homogenate.

As with liver, male kidney catalase was depressed to a greater extent than female. In fact only a very slight decrease in female kidney catalase was observed even after large doses of tumour homogenate.

No effect of either hormones or tumour homogenate on blood catalase was observed. The finding that catalase sensitivity to tumours is only present in those tissues where hormonal influences can be demonstrated may be taken as additional evidence in support of the hypothesis previously put forward (Adams, 1951) that the change of catalase activity produced by tumours is an anti-hormone effect.

### SUMMARY

(1) Liver, kidney, and blood catalase levels have been measured in 14 strains of mice, and in 3 strains of rats. All the male mice had much higher kidney catalase levels than the females, and the males of all strains except 2 (C57BL and C57Br/cd) had higher liver catalase levels than the females. No sex difference was present in blood catalase level. The 3 strains of rats showed a sex difference in liver and kidney catalase levels, but not in blood.

(2) Castration reduced liver and kidney catalase levels in 2 strains of mice examined. Blood catalase was unaffected.

(3) Injection of testosterone into female mice resulted in a rise in catalase level in liver and kidney but not in blood. Kidney catalase took longer than liver catalase to react to testosterone.

(4) Injection of tumour homogenate depressed catalase activity in liver and kidney but not in blood. Liver catalase was affected before kidney catalase. The liver and kidney enzymes in males were affected by tumour homogenate to a greater extent than the corresponding enzymes in females.

My thanks are due to Dr. M. H. Salaman for his interest, to Mr. P. Diamond for skilled technical assistance, and to Mr. J. A. Rawlings for his care of the animals. I am also grateful to the Chester Beatty Research Institute, the National Institute for Medical Research, and the Laboratory Animals Bureau, for their kindness in providing most of the strains of mice and rats used. The expenses of this research were partly defrayed out of a block grant from the British Empire Cancer Campaign.

### REFERENCES.

Adams, D. H.—(1950) *Brit. J. Cancer*, **4**, 183.—(1951) *Ibid.*, **5**, 115.—(1952) *Biochem J.*, **50**, 486.

*Idem* and Berry, M. E.—(1956) *Ibid.*, **64**, 492.

*Idem* and Roe, F. J. C.—(1953) *Brit. J. Cancer*, **7**, 509.

Day, E. D., Gabrielson, F. C. and Lipkind, J. D.—(1954) *J. nat. Cancer Inst.*, **15**, 239.

Greenstein, J P. and Andervont, H. B.—(1942) *Ibid.*, **2**, 345.

*Idem*, Andervont, H. B. and Thompson, J. W.—(1942) *Ibid.*, **2**, 589.

*Idem*, Jenrette, W. V., and White, J.—(1941) *Ibid.*, **2**, 283.

Hargreaves, A. B. and Deutsch, H. F.—(1952) *Cancer Res.*, **12**, 720.

Schultz, M. O. and Kuiken, K. A.—(1941) *J. biol. Chem.*, **137**, 727.